# Supplementary material for: Genetic Overlap Between Alzheimer’s Disease and Depression Mapped Onto the Brain
Source: Front Neurosci. 2021 Jul 5;15:653130. doi: 10.3389/fnins.2021.653130 (PMC8288283; doi:10.3389/fnins.2021.653130)
Supplement: Supplementary file 1 [file Table_1.docx]

***Supplementary Information***

***Supplementary Table 1.***

*List of the 96 brain measures included in the analyses*

| Global measures and subcortical volumes | Regional cortical thickness and surface area |
| --- | --- |
| 1. Estimated intracranial volume | 1.     Bankssts |
| 1. Cerebrospinal fluid | 2.     Caudalanteriorcingulate |
| 1. Total white surface area | 3.     Caudalmiddlefrontal |
| 1. Mean cortical thickness | 4.     Cuneus |
| 1. Lateral Ventricle | 5.     Entorhinal |
| 1. Inferior Lateral Ventricle | 6.     Fusiform |
| 1. Cerebellum White Matter | 7.     Inferiorparietal |
| 1. Cerebellum Cortex | 8.     Inferiortemporal |
| 1. Thalamus Proper | 9.     Isthmuscingulate |
| 1. Caudate | 10.   Lateraloccipital |
| 1. Putamen | 11.   Lateralorbitofrontal |
| 1. Pallidum | 12.   Lingual |
| 1. 3rd Ventricle | 13.   Medialorbitofrontal |
| 1. 4th Ventricle | 14.   Middletemporal |
| 1. Brain Stem | 15.   Parahippocampal |
| 1. Hippocampus | 16.   Paracentral |
| 1. Amygdala | 17.   Parsopercularis |
| 1. Accumbens Area | 18.   Parsorbitalis |
| 1. Ventral Diencephalon | 19.   Parstriangularis |
| 1. Vessel | 20.   Pericalcarine |
| 1. Choroid Plexus | 21.   Postcentral |
| 1. 5th Ventricle | 22.   Posteriorcingulate |
| 1. Optic Chiasm | 23.   Precentral |
| 1. Corpus Callosum Posterior | 24.   Precuneus |
| 1. Corpus Callosum Mid Posterior | 25.   Rostralanteriorcingulate |
| 1. Corpus Callosum Central | 26.   Rostralmiddlefrontal |
| 1. Corpus Callosum Mid Anterior | 27.   Superiorfrontal |
| 1. Corpus Callosum Anterior | 28.   Superiorparietal |
|  | 29.   Superiortemporal |
|  | 30.   Supramarginal |
|  | 31.   Frontalpole |
|  | 32.   Temporalpole |
|  | 33.   Transversetemporal |
|  | 34.   Insula |
